# Role of the Gut–Liver–Kidney Axis in Disease Manifestation and Biomarker Alterations

**DOI:** 10.3390/ijms27188201

**Published:** 2026-09-15

**Authors:** Ryuichi Mashima, Nobuyuki Ishige

**Affiliations:** 1 Department of Clinical Laboratory Medicine, National Center for Child Health and Development, 2-10-1 Okura, Setagaya-ku, Tokyo 157-8535, Japan; 2 Division of Newborn Screening, Tokyo Health Service Association, 1-2-59 Ichigaya-Sadohara, Shinjuku 162-8460, Japan

**Keywords:** metabolome, biomarker, anaerobe, mass spectrometry

## Abstract

Biomarkers with potential for use in clinical diagnosis have been widely studied using biochemical techniques. In particular, endogenous and exogenous metabolites have been targeted in clinical settings. Both types of metabolites are either unmetabolized, metabolized with human enzymes, or metabolized with host-resident bacterial enzymes. In standard clinical chemistry, a specimen must be collected following overnight fasting to minimize the effect of dietary input and the subsequent host and microbial metabolism. Current technology allows the detection of nanomolar concentrations of exogenously derived biomarkers, and these ultra-high sensitivity assays have increased the use of trace amounts of such biomarkers. This narrative review explores how the gut–liver–kidney axis modulates disease manifestation and biomarker accumulations in chronic disorders and inborn errors of metabolism in neonates.

## 1. Introduction

More than 200,000 compounds present in the human metabolome have been registered in the Human Metabolome Database [1]. More specifically, this database contains any components with endogenous or exogenous origins that have been detected in human specimens. Endogenous metabolites include carbohydrates, amino acids, nucleic acids, lipids, and their conjugates, while exogenous compounds include dietary metabolites, pharmaceuticals, and other organic chemicals. Changes in endogenous compounds have been identified as useful markers for the purpose of clinical diagnosis. Among these compounds, both aqueous and hydrophobic compounds are present in a specimen under heterogenous conditions. For example, lipids such as triglycerides, cholesterols, and phospholipids form a lipoprotein in the serum, while other groups of hydrophobic compounds, such as pharmaceutics, bind to albumin in the serum. From a technical perspective, the term “metabolome” usually refers to a set of aqueous compounds, whereas “lipidome” refers to hydrophobic compounds that mostly contain lipids [2,3].

Human metabolomes are most commonly studied using mass spectrometry-based techniques. Thus, such studies are generally performed using either liquid chromatography–tandem mass spectrometry (LC-MS/MS) or liquid chromatography–quadrupole time-of-flight [4,5]. For target analysis, LC-MS/MS is considered to be most appropriate for metabolome quantification. Furthermore, recent studies have measured multiple metabolites using non-target analysis for exploratory purposes. Dietary metabolomics concerns the study of the role of unique diet-derived components detected in humans [6,7]. Recent findings indicate that the quantitative measurement of dietary metabolomics suggests how it works. Such studies routinely measure the concentration of 50–150 compounds in human blood samples. These compounds can be classified into several groups based on their concentrations. A focused metabolome analysis measures the concentration of these compounds, thereby indicating whether the phenotype of interest can be explained by the alteration of the metabolome. Non-target analysis of the metabolome allows identification of previously uncharacterized metabolites in the specimen of interest, which can lead to the discovery of novel biomarkers. In other studies, gas chromatography–mass spectrometry is used. More specifically, for this assay, aqueous components are extracted, silylated, and quantified. Volatile components are collected via microextraction, followed by derivatization for quantification purposes. Supercritical fluid chromatography–tandem mass spectrometry is a recently emerging technique with potential for use in future metabolome research [8,9].

Conjugation is a biochemical process where a metabolite is modified into another substance for excretion. (Table 1). A well-known example of this is sulfate conjugation of steroid hormones [10]. Here, steroid hormones are produced from cholesterol by a variety of hydroxylases. These steroid hormones can be conjugated with sulfate in the liver. The physiological role of this reaction is considered to be temporal inactivation or storage of steroid hormones. When necessary, steroid hormones are regenerated via de-sulfation to maximize the hormonal action at the target site. Similarly, conjugation reactions are used for the regulatory purposes of the local mediator. Inactivation and storage of leukotrienes are mediated by cysteinylation, which regulates the potent inflammatory function [11]. Gut microbe-derived phenolic compounds tend to be conjugated with either sulfate or glucuronate to ensure regulation of neuroinflammatory function [12].

This narrative review mainly focuses on recent publications how the gut–liver–kidney axis affects disease manifestation and biomarker alterations. References were searched on PubMed using a filtering function of a publication date within 5 years. The considered metabolomes include both endogenous and exogenous metabolites that are capable of being generated by either human or gut microbial enzymes. Some old references were cited when an appropriate reference was not found in 2022–2026.

## 2. Microbes in Humans

Aerobic organisms use molecular oxygen as an effective energy source. Most noticeably, such organisms employ superoxide dismutase and catalase [15,16]. Superoxide dismutase reduces superoxide anion O_2_^−^ into water in the cytosol and matrix of mitochondria, while catalase reduces hydrogen peroxide into water in peroxisomes. Anaerobic microbiomes lack these enzymes, which indicates molecular oxygen to be toxic to anaerobic microbes. Similarly, oxygenation only occurs in aerobic organisms; for example, the oxygenation of phenol into catechol by phenol oxidase, cholesterol into steroid for steroid hormones by P450s, and proline into hydroxyproline for collagen synthesis by proline hydroxylase. In contrast, anaerobic microbes catalyze a variety of unique reactions, including decarboxylation and deamination. The addition of methyl groups is another reaction unique to these species, whereas the donation reaction of methyl groups is largely limited to *S*-adenosylmethionine-mediated reaction in aerobes. To a lesser extent, the methylation of homocysteine occurs via methionine synthase using 5-methyltetrahydrofolate as a substrate [17].

*Bacteroides*, *Bifidobacterium*, and *Clostridium* are known obligate anaerobic bacteria, which play a key role in the gut under anaerobic conditions [18,19]. *Clostridium* contains a wide variety of metabolites, while *Bifidobacterium* consists of specific carbohydrates, such as oligosaccharide, food-derived fiber, human milk oligosaccharides, and the sugar chain of glycoproteins. These substrates are metabolized into acetic acid and lactic acid, respectively. Moreover, *Bacteroides* catalyzes oligosaccharides, polysaccharides, mucins, proteins, and amino acids, including branched amino acids. As a result, propionate, acetate, succinate, secondary bile acids, and sphingolipids are produced by *Bacteroides* [20]. In general, the enzymatic reaction of the gut bacteria has been well characterized. Overall, decarboxylation and deamination are well-known reactions that host animals rarely exhibit. The addition of hydrogen atoms to the double bonds of lipids is also catalyzed by *Bacteroides*.

A growing body of evidence demonstrates that several endogenous metabolites are processed by anaerobic bacteria through unique reactions [21]. The best-known example of this involves bile acids, which are essential for the excretion of liver-derived material as a detergent in vivo [21]. Primary bile acids are known to be generated by cholesterol through action with host-derived enzymes, while secondary bile acids are produced by obligate anaerobes. Specifically, deoxycholate and lithocholate are generated by 7α-dehydratase from primary bile acids in response to *Firmicutes*, another type of obligate anaerobe [22]. Ursodeoxycholate is generated by epimerization of 7α-OH by multiple microbiomes involving *Firmicutes*, *Actinobacteria*, and *Pseudomonadota* [22]. These secondary bile acids are also essential for the proper homeostasis of hepatic metabolites. Secondary bile acids normally share 10–20% of the total bile acids; however, they play a distinct role in lipid excretion. Essentially, secondary bile acids lose their conjugation and hydroxyl groups, which dramatically reduces the solubility in water. This physical property conveys a higher affinity for hydrophobic lipids such as triglycerides. Furthermore, some secondary bile acids acquire a higher affinity for the farnesoid X receptor, a lipophilic nuclear receptor, leading to attenuation of cholesterol biosynthesis via *SREBP-1c* gene expression [23,24]. Secondary bile acids may bind to the G-protein-coupled bile acid receptor 1 (GPBAR1), which is also known as the Takeda G-protein-coupled receptor 5, followed by activation of the *UCP-1* gene in adipocytes, leading to body temperature homeostasis [25].

Short-chain fatty acids can be produced by *Firmicutes* and *Clostridium butyricum* through anerobic fermentation of food fibers, such as resistant starch. These short-chain fatty acids can modulate host immune function by means of regulatory T-cells [26], where butyrate inhibits histone deacetylase activity for the *FOXP3* gene, leading to the accelerated differentiation of regulatory T-cells.

## 3. Exogenous Metabolites

Exogenous metabolites obtained from plants and fungi have a unique pool of secondary metabolites [27,28,29,30]. Most dietary metabolites can be classified as exogenous. These secondary metabolites are synthesized by extra enzymes in each species, which explains their wide variety. Table 2 presents some examples of plant- and fungi-derived metabolites. Essentially, plants have polyphenols and the associated glucoside, which are indicative of unique plant-derived dietary features. There are several possible reasons why plants have these specific metabolites. One possibility could be that, in contrast to humans and other animals, plants cannot easily move to avoid from harsh conditions, such as high/low temperature. Thus, plants must synthesize unique metabolites as plant hormones for protection. Another possible reason relates to the notion that some polyphenol is essential for protection against external damage such as ultraviolet radiation, oxidative stress, and viral pathogens. Glycosylation controls the toxicity of polyphenol and increases its water solubility and stability. Hence, from the perspective of homeostatic function, the physiological role of polyphenol glycosylation in plants appears to be similar to that of sulfate conjugation in humans [27,31].

### 3.1. Host Enzyme Metabolites

There are several examples of exogenous metabolites being metabolized by human enzymes. For instance, aflatoxinB1, a natural peanut-derived toxin, is known to be oxidized by the hepatic CYP3A4 and CYP1A2 enzymes to increase its acute hepatic pathogenicity in humans [60]. Furthermore, glucosinolate in broccoli and rapeseed can be converted into heterocyclic amine in the presence of protein by CYP1A2 and N-acetyltransferase 2 under a condition of high temperature, indicating potent carcinogenicity. Aside from these examples that are toxic in humans, the number of reported biomarkers in this category is relatively low.

**Figure 1:**
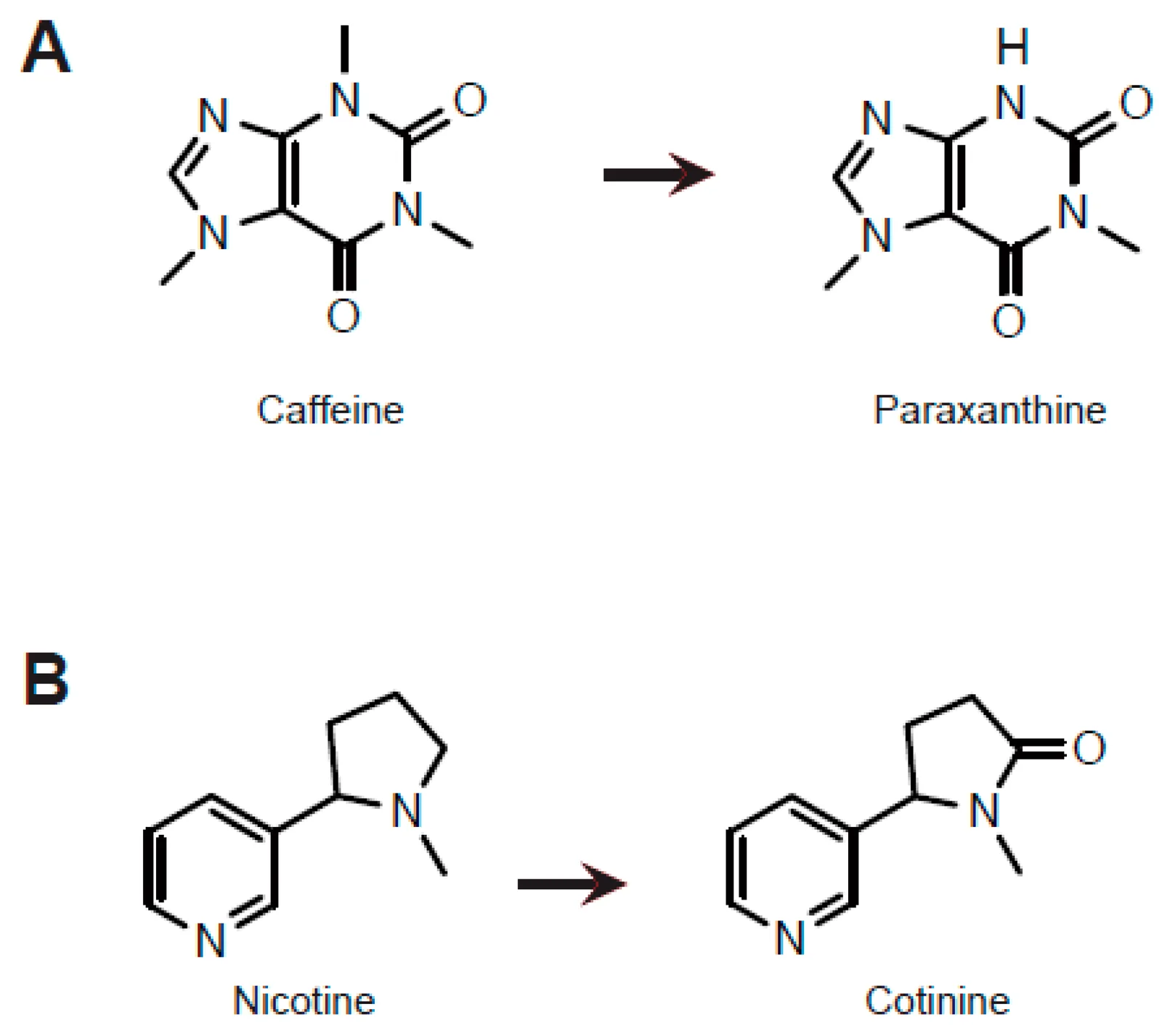
(**A**) Conversion of caffeine into paraxanthine. (**B**) Conversion of nicotine into cotinine.

Caffeine is a well-known exogenous metabolite of plant origin that is mainly metabolized into paraxanthine by CYP1A2 in the liver [61,62] (Figure 1A). Paraxanthine is a 3-*N*-demethylated form of caffeine with longer stability in humans. Thus, the ratio of paraxanthine to caffeine can be used as a measure of the CYP phenotyping marker in conjunction with CYP1A2. Nicotine is another exogenous metabolite, which is incorporated into the body by smoking. Nicotine is metabolized into cotinine by CYP2A6 [63,64] (Figure 1B). Cotinine is a 5′-oxo form of nicotine with a longer half-life. Therefore, cotinine is widely used as a biomarker for total exposure amount of nicotine, including passive smoking, in both clinical and epidemiological studies.

In the case of lysosomal storage disorders, there are examples of plant- or fungi-derived dietary substrates being converted into other metabolites. For example, Fabry disease is a lysosomal storage disorder characterized by the accumulation of globotriaosylsphingosine (LysoGb_3_), an *N*-deacylated form of globotriaosylceramide, in the body [65]. Studies have reported the detection of lysoGb_3_ and its analogs in affected individuals [66,67,68]. By means of a targeted lipidomics approach, it was determined that phytosphingosine-containing Gb_3_ or bacterially metabolized Gb_3_ can be converted into their *N*-deacylated lysoGb_3_ analogs [65].

### 3.2. Bacterial Enzyme Metabolites

Plant-derived metabolites are susceptible to enzymatic degradation in the host-resident gut microbiome (Table 3). In general, multiple enzymes act in coordination to metabolize these substrates. Normally, the polymerized substrate is digested into a monomer in the first group of microbes. Next, the opening of the C-ring in the polyphenol is degraded into small compounds. This reaction occurs in different species of microbes. These biomarkers are useful for the qualitative estimation of precision nutrition [69,70].

## 4. Role of the Gut–Liver–Kidney Axis in Disease Manifestation

Endogenous metabolites are susceptible to being metabolized in host-resident microbes. Among the endogenous metabolites, a limited number have been validated as diagnostic biomarkers. This evidence is supported by the in vivo situation whereby the amount and distribution of host-resident microbes changes under various circumstances. For example, in the case of “opportunistic infection,” the host may be infected by mild external pathogens, particularly when the host immune response is attenuated. Similarly, the levels of biomarkers for a particular disease may be affected by host-derived microbes. Thus, newly proposed biomarkers tend to be used in a panel of biomarkers with similar behavior, especially when a proper validation process has not yet been completed due to the lack of an appropriately isotope-labeled compound.

### 4.1. Metabolic Dysfunction-Associated Steatotic Liver Disease (MASLD)/Metabolic Dysfunction-Associated Steatohepatitis (MASH)

MASLD has emerged as the most prevalent chronic liver disease worldwide, with an increasing number of patients progressing to cirrhosis and hepatocellular carcinoma [79,80]. The progression of MASLD/MASH involves the gut microbiota–bile acid–farnesoid X receptor axis [79], thyroid receptor type-β [81], glucagon-like peptide-1 [82], sodium-glucose cotransporter 2 [83], and other signaling pathways. Among them, gut microbiota–bile acid–farnesoid X receptor axis plays an important role [79]. Modulation of gut microbiome has been examined whether it improves disease manifestations either alone [84] or together with a small molecule drug such as glucagon-like peptide-1 [85].

#### Glycochenodeoxycholate

Glycochenodeoxycholate is produced from chenodeoxycholate by multiple host enzymes, including CYP27A1 and CYP8B1 in the liver. This glycochenodeoxycholate can be de-glycinated back into chenodeoxylcholate by gut enzymes through a reciprocal reaction. An accumulation of glycochenodeoxycholate has been reported in cases of MASLD/MASH in the plasma [86] (Table 4). The impaired excretion function of the liver caused by glycochenodeoxycholate has been suggested as a cause of this accumulation. Glycochenodeoxycholate activates the farnesoid X receptor and GPBAR1 [25]. The activation of these pathways normally ameliorates pathogenic conditions. However, under MASLD/MASH conditions, glycochenodeoxycholate activates sphingosine-1-phosphate receptor 2-mediated G-protein activation in hepatic stellar cells, which results in more advanced pathogenesis of the liver [21,22].

### 4.2. Chronic Kidney Disease (CKD)

Based on the results of metabolomic study, several CKD-specific biomarkers have been proposed [90]. The rationale for this approach involves the accumulation of these metabolites in the kidney due to impaired excretion capacity. In CKD, all the reported disease-associated biomarkers are produced by obligate anaerobes. Their concentration is normally elevated 6–8 h after digestion with an increasing disease severity.

#### 4.2.1. Trimethylamine N-Oxide (TMAO)

**Figure 2:**
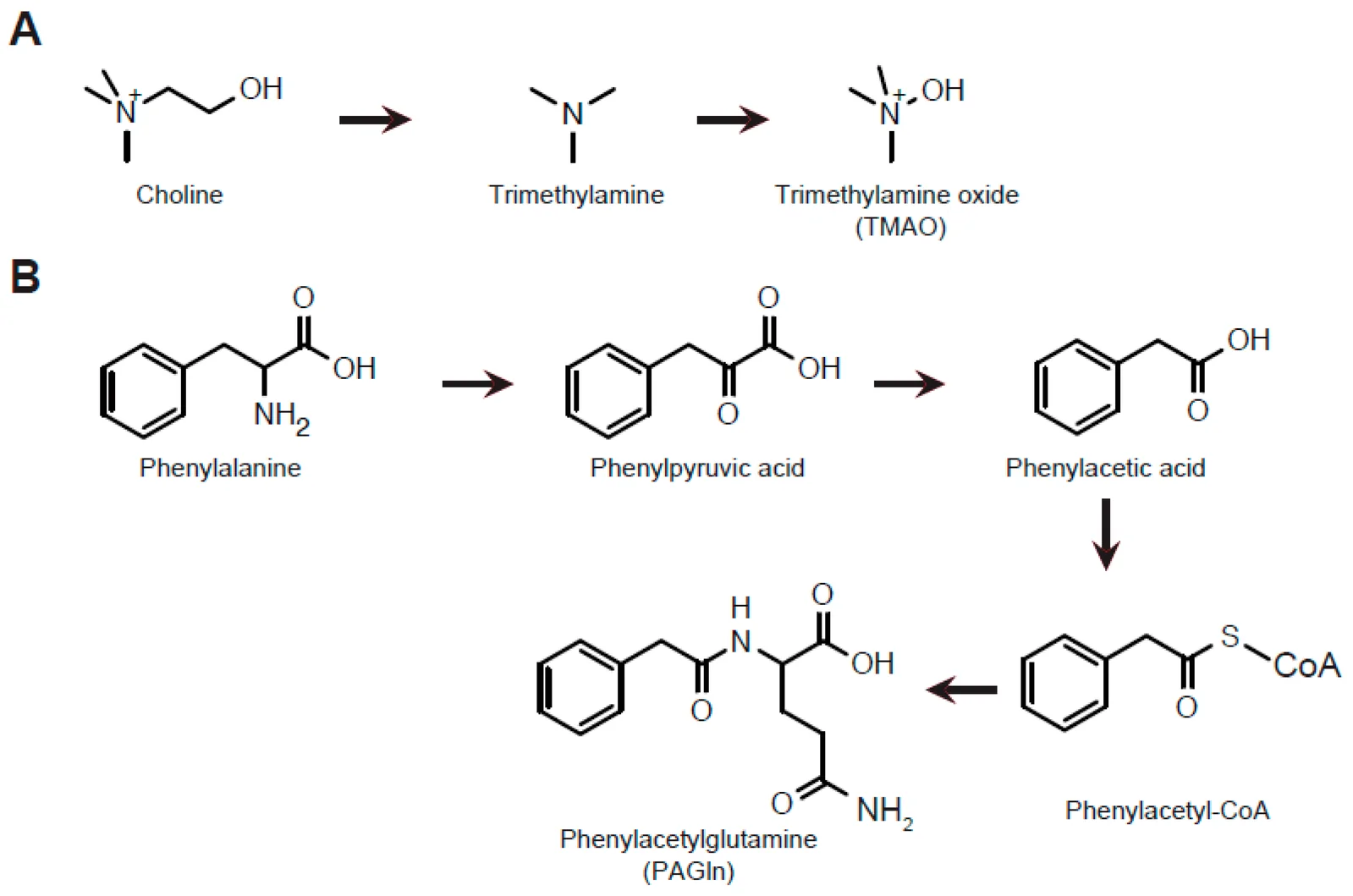
(**A**) Formation of trimethylamine oxide (TMAO) from choline. Choline, either a free form or a variety of modified form such as a phosphodiesterified form in phosphatidylcholine and sphingomyelin, an oxidized form in betaine, and an esterified form in carnitine, is released by a C-N cleavage reaction microbially. Then, the released trimethylamine is oxidized to TMAO by flavin-containing monooxygenase 3 enzyme in humans. (**B**) Formation of phenylacetylglutamine (PAGln) from phenylalanine. Phenylalanine is converted into phenylacetic acid via phenylpyruvic acid by host-resident bacterial enzymes. Subsequently, phenylacetic acid is converted into PAGln via phenylacetyl-CoA by human enzymes.

TMAO is produced by two-step reactions in the host (Figure 2A) [91,92]. First, choline in phosphatidylcholine, L-carnitine, and betaine in meat, eggs, and milk is decomposed into trimethylamine (TMA) and acetaldehyde by means of the choline–TMA lyase enzyme encoded by the *CutC/D* gene of *Firmicutes, Proteobacteria,* and *Actinobacteria* [79]. This TMA is absorbed from the portal vein and then transferred to the liver from the intestine, followed by oxidation by the hepatic flavin-containing monooxygenase 3 enzyme. Normally, TMAO is excreted from the kidney into the circulation. In cases of CKD, TMAO cannot be excreted due to the impaired renal function. Hence, TMAO induces a variety of malfunctions, such as initiation of renal fibrosis, inflammation, and atherosclerosis. Due to TMAO’s toxicity, it has been classified as a uremic toxin [93].

#### 4.2.2. Phenylacetylglutamine (PAGln)

PAGln is a biomarker for CKD with the property of a uremic toxin (Figure 2B) [94]. In this regard, phenylalanine is first converted into phenylacetic acid by a deamination reaction. The newly formed phenylacetic acid is then converted into phenylacetyl-CoA, and this activated intermediate is esterified with glutamine, leading to PAGln. The source of the enzyme responsible for this reaction has been initially identified as *Clostridium* [95].

#### 4.2.3. Indoxyl Sulfate

Indoxyl sulfate is another CKD-specific biomarker metabolized from tryptophan [96,97]. More specifically, tryptophan is metabolized into indole by the tryptophanase enzyme found in *Escherichia coli*. This indole is then converted into indoxyl—namely, 3-hydroxylindole—by the hepatic CYP2E1 enzyme through Phase I reaction. This indoxyl is conjugated with sulfate by the host sulfotransferase family 1A member 1 (SULT1A1) enzyme, leading to indole sulfate. During this reaction, 3′-phosphoadenosine-5′-phosphosulfate (or activated sulfate) acts as a sulfate donor. SULT1A1 is responsible for sulfate conjugation for exogenous compounds; therefore, this gene expression is activated by the aryl hydrocarbon receptor and the pregnane X receptor and constitutive androgen receptor.

### 4.3. Alcoholic Liver Disease

The microsomal ethanol oxidizing system (MEOS) is another ethanol-metabolizing system playing a key role when a large amount of ethanol needs to be metabolized [98,99]. In this reaction CYP2E1 generates an ethoxyl radical and associating reactive oxygen species, leading to enhanced oxidative conditions. The level of CYP2E1 protein is negatively regulated by PAGln, a gut microbiome-mediated metabolite of phenylalanine. The generated reactive oxygen species induce oxidative stress leading to hepatic necrosis. Anti-CYP antibody reacts with CYP2E1 enzyme regulating hepatic pathology.

### 4.4. Herb-Induced Liver Injury (HILI)

HILI is induced by herbs, supplements, natural medicine, and crude drugs, showing toxin-mediated or idiosyncratic liver injury [100,101]. PAGln is a predictive marker for liver disorders from dietary phenylalanine to phenylacetylglutamine by the gut microbe. PAGln exacerbates HILI with three distinct mechanisms. First, vascular endothelial cells can be activated by PAGln by oxidative stress. Second, PAGln stimulates platelet activation followed by hepatic necrosis and ischemic damage. Third, an increase in PAGln concentration activates immune cells, leading to an inflammatory cytokine release. Thus, an increase in individuals with PAGln has been suggested as an occurrence of HILI with high incidence [102].

### 4.5. Drug-Induced Liver Injury (DILI)

DILI is drug-induced liver injury caused by both synthesized drugs and herbal medicine without any association with viruses and alcohol [103,104,105,106]. Roussel Uclaf Causality Assessment Method (RUCAM) updated in 2016 is a diagnostic tool to verify the correlation between manifestation and DILI with a clinically structured and quantified scoring system [107]. This new RUCAM evaluates this correlation with −9 to 14 points with five grades. More specifically this RUCAM has four major quantitative criteria. First, a hepatic injury is classified with hepatocellular injury, cholestatic injury, and mixed injury based on the biochemical level of ALT and ALP. Second, the rate of dechallenge was quantitatively monitored. Third, other causative factors such as hepatitis types A–E, viral infection, autoimmune hepatitis, and biliary obstruction were excluded. Fourthly, the effect of rechallenge with other drugs was examined. In contrast to this established RUCAM in 2016, DILI-specific biomarkers have not been validated. This reason could be attributed to the following three reasons. First, the manifestation of idiosyncratic DILI is not modeled with any known animal models. Second, currently proposed novel biomarkers, such as miRNA-122, high mobility group box 1, keratin 18, and glutamate dehydrogenase, only show low specificity for DILI. Third, a lack of solid diagnostic tools for DILI requires confirmed RUCAM-high individuals to test the potential biomarker. Although much effort has been made to establish a biomarker for DILI, none has been established now.

## 5. Role of the Gut–Liver–Kidney Axis in Biomarker Alterations in Newborn Screening

Newborn screening is performed to identify inborn metabolism errors in infants [108,109]. Phenylketonuria and medium-chain acyl-CoA dehydrogenase deficiency are two disorders with more than five decades of screening history. While these disorders were initially screened via microbiological techniques, an MS/MS-based assay is widely used today. The advantages of the latter technique include quantification with multiple reaction monitoring, which provides greater efficiently. In fact, current newborn screening programs examine nearly 20 disorders using a single punch taken from a dried blood spot.

### 5.1. Isovaleric Acidemia

**Figure 3:**
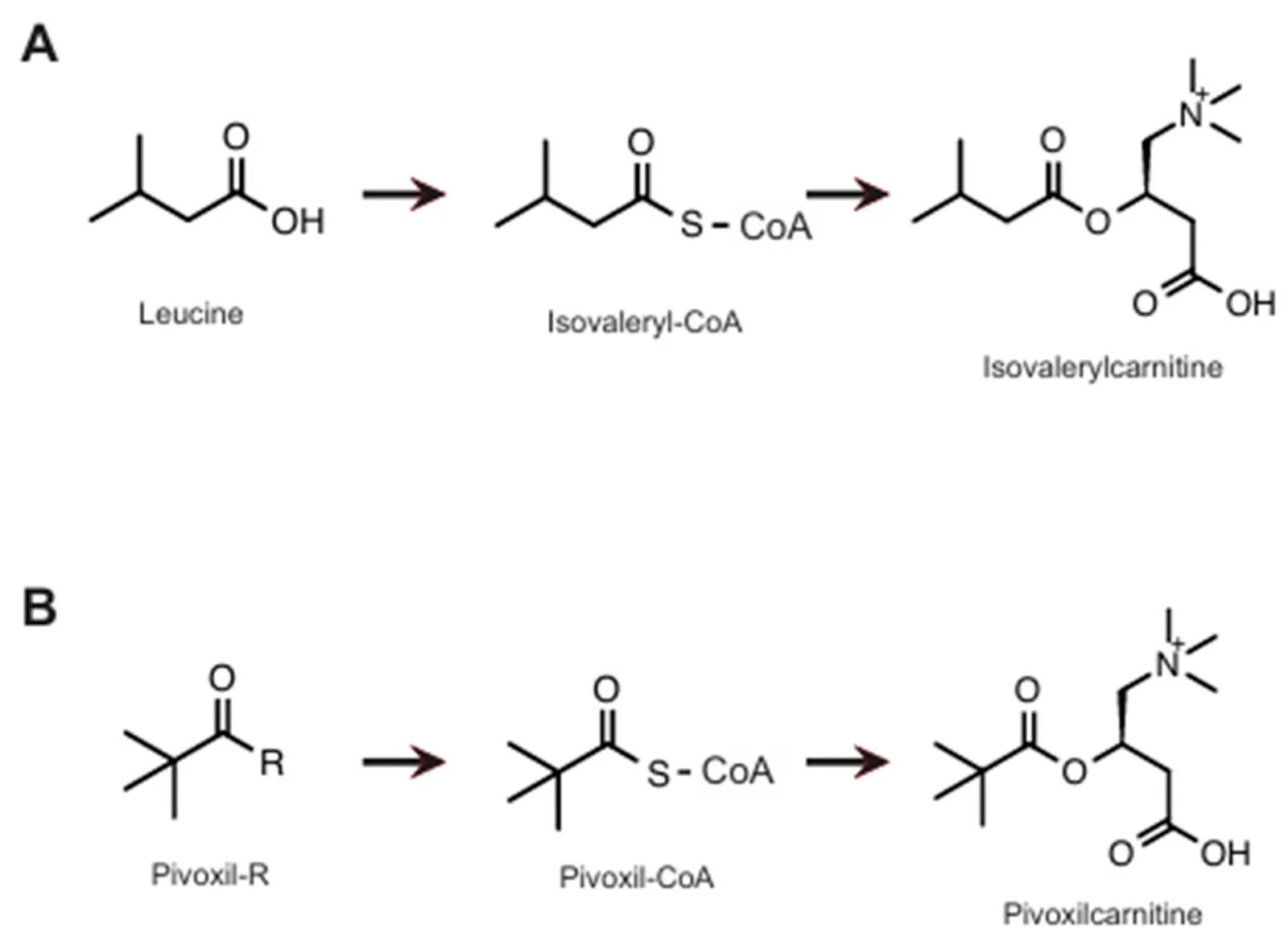
(**A**) Conversion of leucine to isovalerylcarnitine via isovaleryl-CoA. (**B**) Conversion of pivoxil-containing antibiotics to pivoxilcarnitine.

Isovaleric acidemia is an inborn error of metabolism characterized by the accumulation of isovaleric acid in the blood due to impaired leucine degradation [110] (Figure 3A). The major manifestations of isovaleric acidemia include hyperammonemia and neurologic symptoms such as tremors and seizures. The metabolism of leucine in humans primarily occurs in the mitochondria by means of isovaleryl-CoA dehydrogenase. Importantly, leucine can also be metabolized by antibiotic-sensitive enzymes in gut microbes such as *Clostridium*, *Bacteroides*, *Peptostreptococcus*, and *Propionibacterium.* Thus, treatment of neonates with antibiotics can lead to false-positive results in terms of newborn screening [110,111,112,113] (Figure 3B).

### 5.2. Mucopolysaccharidosis Type II

Mucopolysaccharidosis is a lysosomal storage disorder characterized by the accumulation of glycosaminoglycans in the body [114]. Moreover, heparan sulfate (HS) consists of multiple units of glucuronic acid and *N*-acetylglucosamine disaccharide. A defect in the iduronate-2-sulfatase enzyme causes mucopolysaccharidosis type II, which is why HS is regarded as a good biomarker for its diagnosis [115]. For the purpose of quantification, a unit of iduronate–glucosamine enzymatically generated using a mixture of heparinases from the aerobic microbe *Flavobacterium heparinum* has previously been investigated. Recently, a newly identified fragment of HS has been applied in the diagnosis of mucopolysaccharidosis type II [116,117]. The origin of this HS fragment in humans is not well-documented at this stage. In a separate study involving anerobic microbes, it has been determined that HS is metabolized by *Bacteroides thetaiotaomicron* and related bacterial species [118,119]. Intact HS and heparin are oligomerized by a hydrolase (BT4462) at the outer membrane of the *Bacteroides* and then incorporated into cytosol through a translocator complex (BT4659-4661). These oligomers are de-sulfated by 2-*O*-sulfatase (BT1596) and a number of hydrolases (BT4656, 4652, 4675). Next, the monomeric unit of glucuronic acid and *N*-acetylglucosamine produced is hydrolyzed into a glucuronic acid and an *N*-acetylglucosamine by the glycoside hydrolase GH88 (BT4658). The amount of *Bacteroides* in humans is higher than in other species, particularly in the large intestine, which explains why these enzymes are thought to be responsible for the accumulation of HS fragments in humans in both physiological and pathophysiological settings. The newly identified HS fragment was also detected in the liver and kidney in a mucopolysaccharidosis type II mouse model [120].

### 5.3. Role of Biomarkers in Expanded Newborn Screening

Newborn screening is currently performed using LC-MS/MS for amino acid disorders, organic acid disorders, and lysosomal storage disorders [108,121,122,123]. More recently, to include immunodeficiency with a T-cell receptor excision circle (TREC) and spinal muscular atrophy (SMA) as emerging screening candidate disorders, quantitative polymerase chain reaction has been deployed. Given the increase in newly developed therapies for neonatal disorders over time, the demand for screening has also dramatically increased. To ensure future expansion in this regard, genome-based technology is being actively explored today [124,125,126,127,128,129,130,131,132,133,134,135,136,137,138]. This technology allows extended screening with uncharacterized biomarkers. Based on preliminary data, the conditions selected for the ongoing pilot study must satisfy several requirements. First, the currently recommended uniform screening panel (RUSP)-accepted conditions must be included. Second, actionable treatment needs to be established for conditions to be screened. The current RUSP-accepted conditions for newborn screening include metabolic disorders, lysosomal storage disorders, TREC, and SMA. Newborn genome screening also includes—but is not limited to—hematologic disorders, cardiac disorders, and inflammatory disorders. In addition to this streamlined technique, research on biomarkers for use in newborn screening and genome screening is expected to accelerate.

## 6. Conclusions

Clinically relevant disease-specific biomarkers have been studied for more than a century now. Most metabolites have been biochemically characterized during that time. While the associated biochemical reaction has been well-documented, the bacteria responsible have only been partially identified. Yet, due to the increasing number of studies concerning gut bacteria that use a recently developed anaerobic experimental instrument, more precisely characterized biochemistry in anaerobes has been achieved. The changes in each metabolite, whether of endogenous or exogenous origin, can easily be quantified via a streamlined MS-based technique, providing a more complete picture of the metabolome in humans. It should be noted that due to bacterial effects, the disease manifestations and cut-off value for diagnosis may be affected by exogenous events such as alterations of the bacterial composition of the gut flora.

## Figures and Tables

**Table 1:** Primary and secondary metabolites in humans.

Starting Material	Primary Metabolite	Enzyme for Primary Metabolite	Secondary Metabolite	Enzyme for Secondary Metabolite	Site of Production (Organ)	Function of Secondary Metabolite	Ref
Cholesterol	Dehydroepiandrosterone	CYP11A1, CYP17A1	DHEA-sulfate conjugate	SULT2A1	Adrenal cortex, brain	Act as a neurosteroid in the brain. Binds to GABA_A receptor and NMDA receptor, involving memory improvement and control of anxiety.	[13]
Cholesterol	Primary cholic acid)	CYP7A1	Glycine-, taurine-conjugated bile acids	Bile acid CoA: amino acid N-acyltransferase	Liver	Insulin secretion, regulation of energy metabolism, immunosuppression.	[14]
Arachidonate	Leukotriene A4	5-Lipoxygenase	Cysteinyl-leukotriene (LTC4/D4/E4)	LTC4 synthase	Immune cells, such as macrophages and mast cells.	Potent chemical mediator for bronchial contraction and vascular permeability.	[11]
Tyrosine	Thyroxine, triiodothyronine	Thyroid peroxidase	Thyroid hormone sulfate-/glucuronate conjugate	Sulfate transfer enzyme, UDP-glucuronate transferase	Liver, peripheral tissue	Functions as a pool for storage. Can regulate thyroid metabolism upon de-conjugation.	[12]

DHEA, dehydroepiandrosterone; LT, leukotriene; SULT2A1, sulfotransferase family 2A member 1; UDP, uridine diphosphate.

**Table 2:** Typical dietary metabolites in foods.

Origin	Origin	Compound Name	Chemical Species	Note	Ref
Plant	Apple	Phlorizin	Polyphenol	Polyphenol glycoside	[32]
	Broccoli	Glucoraphanin	Glucosinolate	S-containing secondary metabolite	[33]
	Cacao	Theobromine	Alkaloid	Caffeine metabolite	[34]
	Celery, parsley	Apigenin	Polyphenol	Flavone	[35]
	Citrus, alfalfa	Homostachydrine	Betaine	Betaine homolog	[36]
	Citrus (orange, grapefruit)	Stachydrine	Betaine	Proline-derived betaine	[37]
	Citrus peel	Hesperidin	Polyphenol	Polyphenol-glycoside	[38]
	Coffee, burdock root	Chlorogenic acid	Polyphenol	Phenylpropanoid	[39]
	Coffee, tea	1,3,7-Trimethyluric acid	Alkaloid	Caffeine metabolite	[40]
	Garlic	S-Allyl-L-cysteine	Amino acid	S-containing amino acid	[41]
	Garlic, onion	Isoalliin	Amino acid	S-containing amino acid	[42]
	Grape	Resveratrol	Polyphenol	2-Ring compound	[43]
	Grape	Tartaric acid	Organic acid	L-Ascorbic acid metabolite	[44]
	Horseradish, wasabi	Isothiocyanates	Glucosinolate	Organosulfur compound	[45]
	Oats	Avenanthramide	Phenolic amide	Antioxidant	[46]
	Soy	Genistein, Daidzein	Polyphenol	Isoflavone, glycoside	[47]
	Strawberry, berry	Gentisic acid	Phenol	Salicylic acid metabolite	[48]
Animal	Beef, pork	Carnosine	Dipeptide	β-alanyl-L-histidine	[49]
	Chicken, fish	1-Methylhistidine	Amino acid	Exogenous marker	[50]
	Chicken, fish	Anserine	Dipeptide	β-alanyl-1-methylhistidine	[51]
	Dairy product	Pentadecanoate	Lipid	Milk fat	[52]
	Marine seafood	TMAO	Amine oxide	Osmolyte	[53]
	Meat (beef, pork, poultry/chicken)	3-Methylhistidine	Amino acid	Endogenous marker	[54]
	Shellfish (prawn, crab)	Arsenobetaine	Organic arsenite	Arsenic analog of glycine betaine	[55]
Fungi and Ferment	Mushroom	Ergothioneine	Amino acid metabolite	Methionine metabolites	[56]
	Cheese, wine, natto	Histamine	Amino acid metabolite	Histidine metabolite	[57]
	Cheese, wine, natto	Tyramine	Amino acid metabolite	Tyrosine metabolite	[58]
	Fungi, some sea weeds	Mannitol	Polyol	Sugar metabolite	[59]

TMAO, Trimethylamine-N-oxide.

**Table 3:** Microbial-mediated phytometabolites found in humans.

Foods	Group	Substrate	Metabolite	Ref
Soybean	Isoflavone	Daidzein	Equol	[71]
Pomegranate, berries, walnut	Ellagitannin	Ellagic acid	Urolithin A/B	[72]
Sesami, flaxseed, whole grain	Lignan	Sesaminol	Enterolactone	[73]
	Polyphenol	Matairesinol	Enterodiol	[74]
Green tea, apple, cocoa	Flavanol	Catechin	γ-Valerolactones	[75]
	Condensed tannin	Proanthocyanidin	γ-Valerolactones	[76]
Blue berry, red cabbage	Anthocyanin	Cyanidin glycosides	Gallic acid, protocatechuic acid (phenolic acids)	[77]
Broccoli sprouts	Glucosinolate	Glucoraphanin	Sulforaphane	[78]

**Table 4:** Reported biomarkers for chronic kidney disease and steatosis in humans.

Disease	Compound	Production Mechanism	Clinical Significance	Receptor	Reference
MASLD/MASH	Glycochenodeoxycholic acid	Bacteria-specific modification of bile acid	A recently proposed biomarker for steatosis	FXR, GPBAR1	[86]
Chronic kidney disease	Phenylacetylglutamine	Phenylalanine-derived bacterial metabolite	An accumulation in renal excretion failure; a risk in cardiovascular disease	α2A, α2B, β2 adrenaline receptor	[87,88]
	TMAO	Bacterial oxidation of choline followed by hepatic oxidation	Hepatic accumulation of lipids; a direct inducer of inflammation	PKR	[88]
	Indoxyl sulfate	Tryptophan-derived bacterial metabolite	A potent uremic toxin; facilitates renal fibrosis	AhR	[87,88]
	p-Cresyl sulfate	Tyrosine-derived bacterial metabolite	Contribution to blood vessel damage and renal failure development	Not identified	[87,88]
	Kynurenine	Tryptophan-derived metabolite	An association with immune response; an increase in renal failure	AhR	[89]

AhR, Aryl hydrocarbon receptor; FXR, farnesoid X receptor; GPBAR1, G-protein-coupled bile acid receptor 1; PKR, protein kinase RNA-like endoplasmic reticulum kinase.

## Data Availability

Data are provided in the list of references.
